# A critical period for learning and plastic changes at hippocampal CA1 synapses

**DOI:** 10.1038/s41598-022-10453-z

**Published:** 2022-05-03

**Authors:** Yuya Sakimoto, Ako Shintani, Daiki Yoshiura, Makoto Goshima, Hiroyuki Kida, Dai Mitsushima

**Affiliations:** 1 Department of Physiology, Yamaguchi University Graduate School of Medicine, Ube, 755-8505 Japan; 2 The Research Institute for Time Studies, Yamaguchi University, Yamaguchi, 753-8511 Japan

**Keywords:** Neurophysiology, Cognitive neuroscience, Synaptic plasticity

## Abstract

Postnatal development of hippocampal function has been reported in many mammalian species, including humans. To obtain synaptic evidence, we analyzed developmental changes in plasticity after an inhibitory avoidance task in rats. Learning performance was low in infants (postnatal 2 weeks) but clearly improved from the juvenile period (3–4 weeks) to adulthood (8 weeks). One hour after the training, we prepared brain slices and sequentially recorded miniature excitatory postsynaptic currents (mEPSCs) and inhibitory postsynaptic currents (mIPSCs) from the same hippocampal CA1 neuron. Although the training failed to affect the amplitude of either mEPSCs or mIPSCs at 2 weeks, it increased mEPSC, but not mIPSC, amplitude at 3 weeks. At 4 weeks, the training had increased the amplitude of both mEPSCs and mIPSCs, whereas mIPSC, but not mEPSC, amplitude was increased at 8 weeks. Because early-life physiological functions can affect performance, we also evaluated sensory–motor functions together with emotional state and found adequate sensory/motor functions from infancy to adulthood. Moreover, by analyzing performance of rats in multiple hippocampal-dependent tasks, we found that the developmental changes in the performance are task dependent. Taken together, these findings delineate a critical period for learning and plastic changes at hippocampal CA1 synapses.

## Introduction

The hippocampus plays a central role in the formation of episodic memory^1^, processing spatio-temporal information^2,3^ related to a specific event^4,5^. Because postnatal experience enhances the development of the hippocampus, the function is immature in infants^6–10^.

Miles^11^ first published evidence that human adults could not recall specific events from early childhood, which is termed infantile amnesia^7–10^. In rats, Campbell and Campbell^6^ showed rapid forgetting of contextual memory on postnatal day 18. In turn, the retention was clearly improved if the task was performed on postnatal days 38, 54, and 100. Postnatal-day-18 rats consistently showed this deficit^6,12^, with improvement in their performance observed after postnatal day 23^12,13^. However, the learning behavior should be evaluated precisely, because insufficient sensory or motor performance could affect task performance.

Synaptic contacts in hippocampal CA1 neurons continuously increase in the first several weeks of life in rats^14–16^. Spine density dramatically increases from postnatal days 7 to 28, especially from days 7 to 21, expanding by threefold^17^. Moreover, the magnitude of long-term potentiation (LTP), which is a cellular substrate for learning and memory, dramatically increases from postnatal days 10 to 20, with the increase persisting up to day 60^18^. A recent brain-wide analysis of synapses across the lifespan of rats further demonstrated that the molecular/morphological diversity of excitatory synapses was dramatically increased within the first postnatal month, especially in the hippocampus^19^.

Insertion of synaptic membrane AMPA receptors is a major mechanism underlying LTP expression in CA1 pyramidal neurons^20,21^. Regarding the causal relationship, hippocampal-dependent contextual learning not only requires synaptic delivery of AMPA receptors, but also strengthens GABA_A_ receptor-mediated inhibitory synapses onto neurons^22,23^. Moreover, this learning strengthens both excitatory and inhibitory synapses in different ways in individual CA1 neurons, thus producing a broad variety of synaptic input across cells in juvenile males^24,25^. Using the same experimental protocol, here we evaluated a critical change in the performance together with the training-induced plastic change that occurs from infancy to adulthood.

## Results

**Figure 1:**
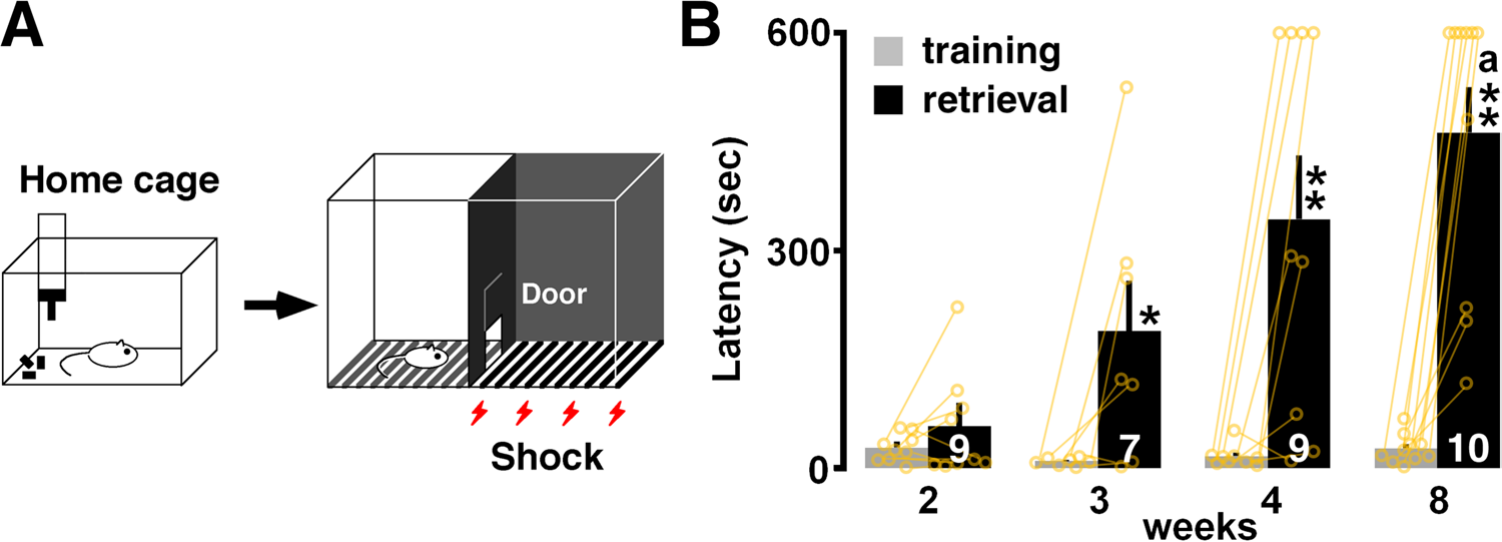
Inhibitory avoidance (IA) task and postnatal development of performance. (**A**) On the training day, we moved the rats from their home cage to the light box. (**B**) Thirty minutes after the training, a longer latency to enter the dark side of the box was observed at 3–8 weeks. The number of rats is shown at the bottom of each bar. Data are shown as individual points and are the mean ± SEM. *P < 0.05 vs. training. **P < 0.01 vs. training. ^a^*P* < 0.05 vs. 2 weeks.

Rats were subjected to the inhibitory avoidance (IA) task (Fig. 1A). They crossed from a light box to a dark box, where an electric foot shock (1.6 mA, 2 s) was delivered. Half an hour after the task, we measured the latency in the illuminated box as contextual learning performance. Figure 1B shows the latency in the training session and the retrieval test. Two-way ANOVA revealed a significant interaction (*F*_3,33_ = 4.73, *P* = 0.0075) and a main effect of developmental stage (*F*_1,33_ = 55.22, *P* < 0.0001) and training (*F*_3,33_ = 3.922, *P* = 0.0169). The training failed to increase the latency at 2 weeks (Fig. 1B).

**Figure 2:**
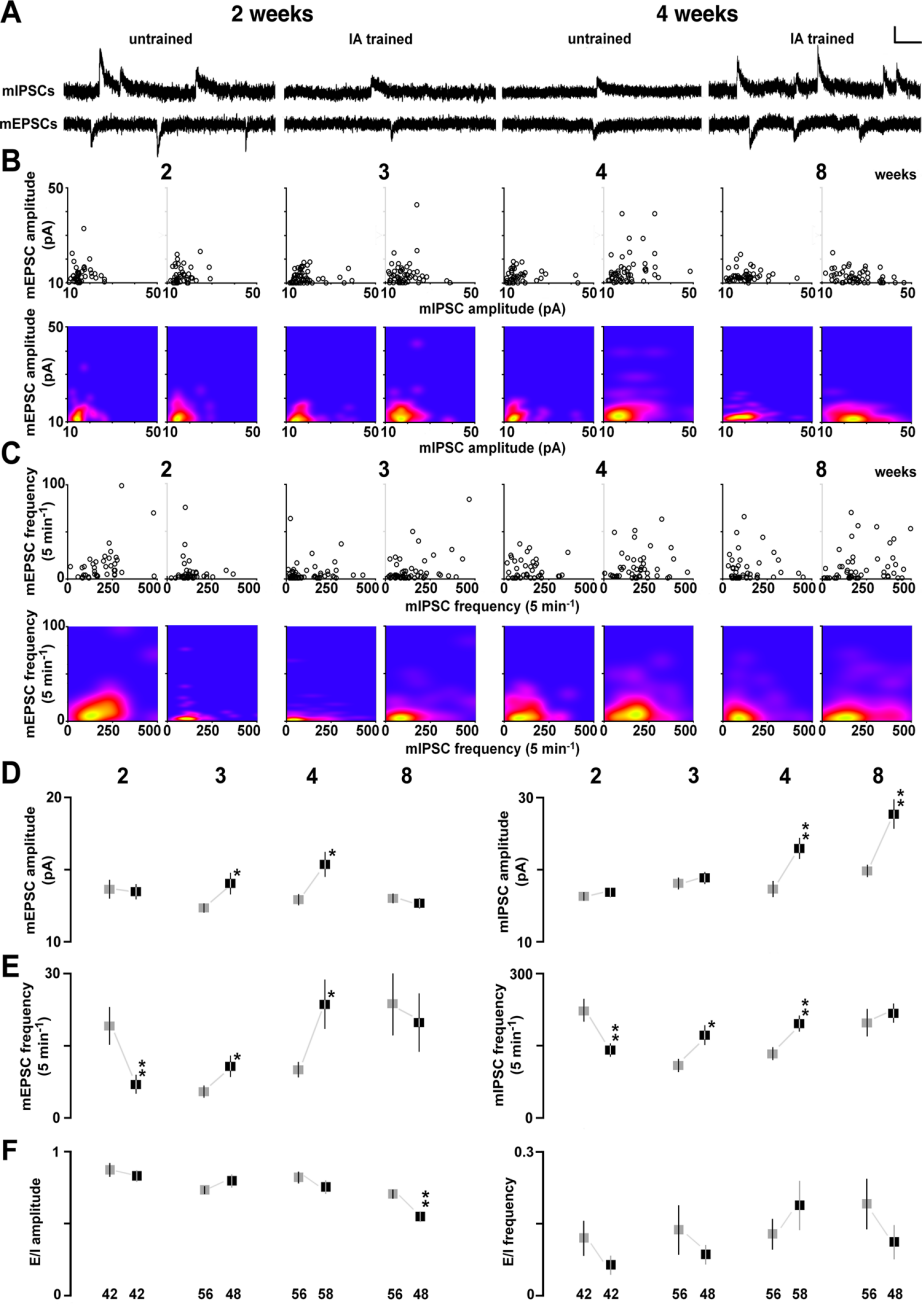
Postnatal development of the IA-task-induced synaptic plasticity. (**A**) Representative traces of mEPSCs and mIPSCs sequentially recorded in the same CA1 pyramidal neuron in the presence of tetrodotoxin (0.5 µM). Vertical bar = 20 pA; horizontal bar = 50 ms. (**B**) Two-dimensional plots of the mean mEPSC and mIPSC amplitudes in an individual neuron (upper panel), and results of the kernel density analysis visualizing the distribution of the appearance probability at any point (lower panel). (**C**) Two-dimensional plots of the mean mEPSC and mIPSC frequencies in an individual neuron (upper panel), and visualization of the kernel density distribution (lower panel). (**D**) Postnatal changes in the mean mEPSC (left) and mIPSC (right) amplitudes in untrained (gray) and trained (black) rats. The IA task increased mEPSC amplitude at 3 and 4 weeks and increased mIPSC amplitude at 4–8 weeks. (**E**) Postnatal changes in the mean mEPSC (left) and mIPSC (right) frequencies in untrained (gray) and trained (black) rats. The training increased the frequencies of both mEPSCs and mIPSCs at 3 and 4 weeks, and decreased these frequencies at 2 weeks. The numbers under the graphs indicate the number of neurons. The error bars indicate ± SEM. *P < 0.05, **P < 0.01 vs. untrained.

To analyze training-induced synaptic plasticity, we recorded miniature excitatory postsynaptic currents (mEPSCs) or miniature inhibitory postsynaptic current (mIPSCs) in the presence of 0.5 µM tetrodotoxin in the dorsal hippocampus (Fig. 2A). By changing the membrane potential, we sequentially recorded mEPSCs (at − 60 mV) and mIPSCs (at 0 mV) from the same neuron, as reported previously^23^. We confirmed that the mEPSC and mIPSC events were clearly blocked by bath treatment with an AMPA receptor blocker (CNQX) or a GABA_A_ receptor blocker (bicuculline). The postsynaptic currents are thought to correspond to the response elicited by a single vesicle of glutamate or GABA^26^. In contrast, the number of synapses affects the frequency of events.

The strength of AMPA receptor-mediated excitatory input vs. GABA_A_ receptor-mediated inhibitory input was measured in each neuron and plotted two dimensionally (amplitude in Fig. 2B, upper panel; frequency in Fig. 2C, upper panel). Regarding the mEPSCs, IA training significantly increased their mean amplitude at 3 and 4 weeks (3 weeks: *t*_102_ = − 2.17; *P* = 0.0323; 4 weeks: *t*_92_ = − 2.57; *P* = 0.0120, unpaired *t*-test; Fig. 2D, left) and their frequency at 3 and 4 weeks (3 weeks: *t*_102_ = − 2.05; *P* = 0.0435; 4 weeks: *t*_92_ = − 2.53; *P* = 0.0140, unpaired *t*-test; Fig. 2E, left). Conversely, the mEPSC frequency was significantly decreased at 2 weeks (*t*_82_ = 2.77; *P* = 0.0070, unpaired *t*-test; Fig. 2E, left). Regarding the mIPSCs, IA training significantly increased their mean amplitude at 4 and 8 weeks (4 weeks: *t*_92_ = − 3.06; *P* = 0.0029; 8 weeks: *t*_103_ = − 3.56; *P* = 0.0006, unpaired *t*-test; Fig. 2D, right) and their frequency at 3 and 4 weeks (3 weeks: *t*_110_ = − 2.56; *P* = 0.0112; 4 weeks: *t*_92_ = − 2.82; *P* = 0.0059, unpaired *t*-test; Fig. 2E, right). Conversely, mIPSC frequency was significantly decreased at 2 weeks (*t*_82_ = 3.01; *P* = 0.0036, unpaired *t*-test; Fig. 2E, right). We also prepared the cumulative distribution in Supplementary Fig. 1.

Here we examined training-induced synaptic plasticity at multiple postnatal ages. Further yoked controls exposed to a foot shock (unpaired) or the apparatus alone (walk through) are necessary to clarify whether the plasticity is induced by learning^23^.

To calculate the balance between excitatory/inhibitory (E/I) input, we divided the mean mEPSC amplitude by the mean mIPSC amplitude in individual neurons. Although the training did not change the E/I balance at 2 (*P* = 0.51), 3 (*P* = 0.32), and 4 (*P* = 0.30) weeks, it clearly decreased the balance at 8 weeks exclusively (*P* = 0.0016; Fig. 2F).

**Figure 3:**
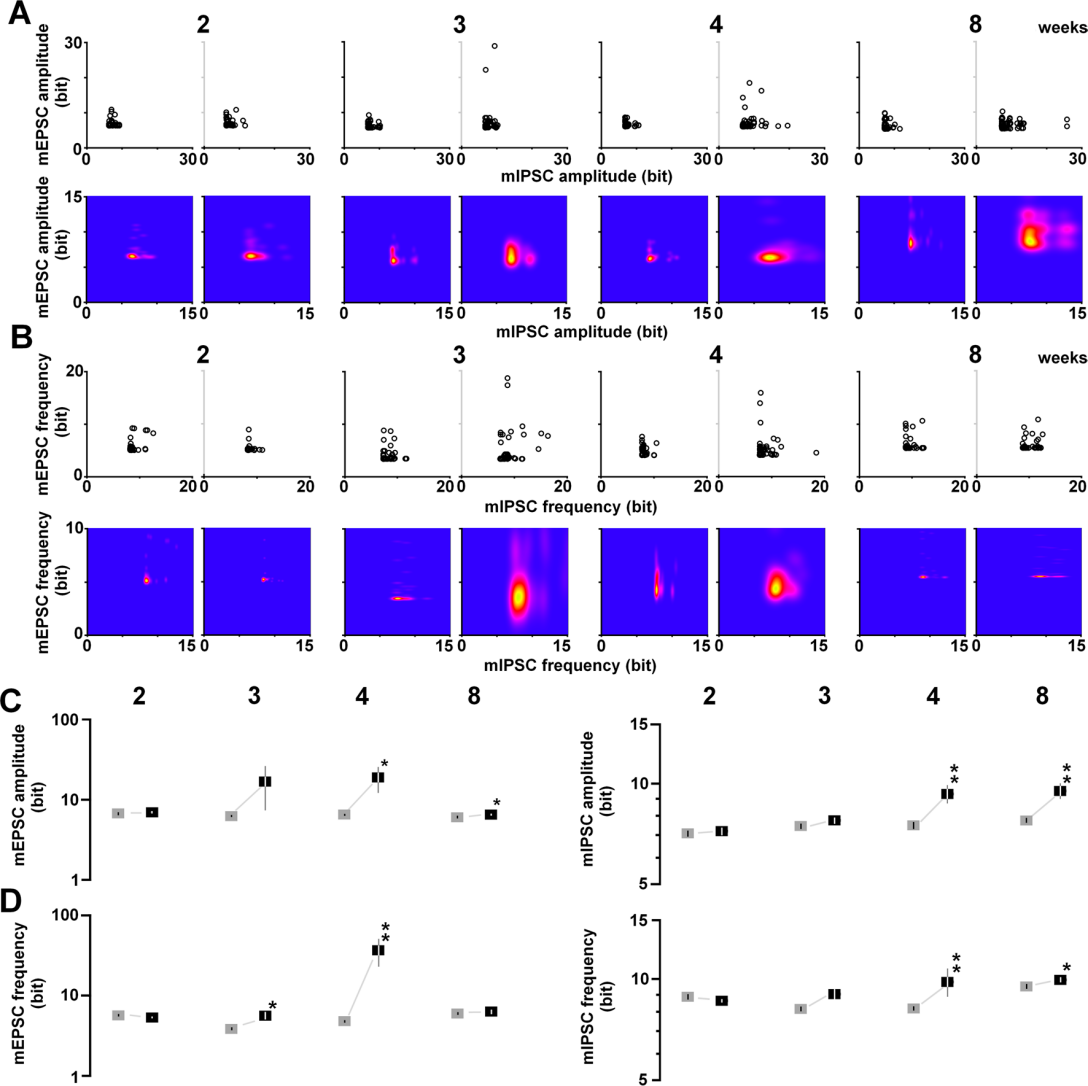
Postnatal development of the gained self-entropy in individual neurons. (**A**) By calculating the appearance probability of individual dots in mEPSC and mIPSC amplitudes, we calculated the self-entropy (bits) of individual neurons (upper), and visualized the kernel density distribution (lower). (**B**) The same process as in (**A**), applied for mEPSC and mIPSC frequencies, showing the self-entropy (bit) of individual neurons (upper) and the visualized kernel density distribution (lower). (**C**) Postnatal changes in the mean self-entropy of mEPSC (left) and mIPSC (right) amplitudes in untrained (gray) and trained (black) rats. The IA task increased the self-entropy at 4–8 weeks. A base 10 log scale is used for the Y axis. (**D**) Postnatal changes in the mean self-entropy of mEPSC (left) and mIPSC (right) frequencies. The IA task increased the self-entropy of mEPSCs (left) at 3–4 weeks, and that of mIPSCs (right) at 4–8 weeks. The numbers under the graphs indicate the number of neurons. The error bars indicate ± SEM. *P < 0.05, **P < 0.01 vs. untrained.

Based on the information theory of Shannon, we calculated the appearance probability of the mean amplitudes of mEPSCs and mIPSCs. First, we identified the distribution of the appearance probability in untrained control animals (Fig. 3A, left), followed by the analysis of the cell‐specific appearance probability of all recorded neurons individually (Fig. 3A, right). Each probability of a single neuron was calculated as the self‐entropy and plotted two‐dimensionally; e.g., a point with a high appearance probability (around the mean level of mEPSC and mIPSC amplitudes) indicated a low self‐entropy, whereas a point with a very rare probability (deviated from mEPSC and mIPSC amplitudes) indicated a high self‐entropy.

We used a two‐dimensional kernel analysis to visualize synaptic density (Fig. 3A, lower panels). IA training clearly diversified the amount of information per neuron, which was sustained. Regarding the mEPSC and mIPSC amplitude, the training significantly increased the self-entropy at 4 and 8 weeks, but not at 2 and 3 weeks (mEPSC, 2 weeks: *t*_82_ = − 0.53; *P* = 0.5915; 3 weeks: *t*_102_ = − 1.82; *P* = 0.0745; 4 weeks: *t*_92_ = − 2.43; *P* = 0.0186; 8 weeks: *t*_103_ = − 2.33; *P* = 0.0220, Fig. 3C, left; mIPSC, 2 weeks: *t*_82_ = − 0.39; *P* = 0.6989; 3 weeks: *t*_102_ = − 1.49; *P* = 0.1384; 4 weeks: *t*_92_ = − 3.49; *P* = 0.0008; 8 weeks: *t*_103_ = − 3.77; *P* = 0.0003, Fig. 3C, right).

For the mEPSC and mIPSC frequency, we identified the distribution of appearance probability in untrained controls (Fig. 3B, left), followed by the analysis of the appearance probability of all recorded neurons individually. We found cell‐specific self‐entropy in all recorded neurons, with self‐entropy varying from cell to cell (Fig. 3B, upper panels).

A two‐dimensional kernel analysis allowed the visualization of synaptic density (Fig. 3B, lower panels). IA training diversified the amount of information per neuron, which was sustained. In the mEPSC frequency, IA training significantly increased the self-entropy at 3 and 4 weeks (3 weeks: *t*_102_ = − 2.60; *P* = 0.0116; 4 weeks: *t*_92_ = − 2.69; *P* = 0.0094, Fig. 3D, left). In the mIPSC frequency, the training significantly increased the self-entropy at 4 and 8 weeks (4 weeks: *t*_92_ = − 3.49; *P* = 0.0008; 8 weeks: *t*_103_ = − 3.77; *P* = 0.0003, Fig. 3D, right).

**Figure 4:**
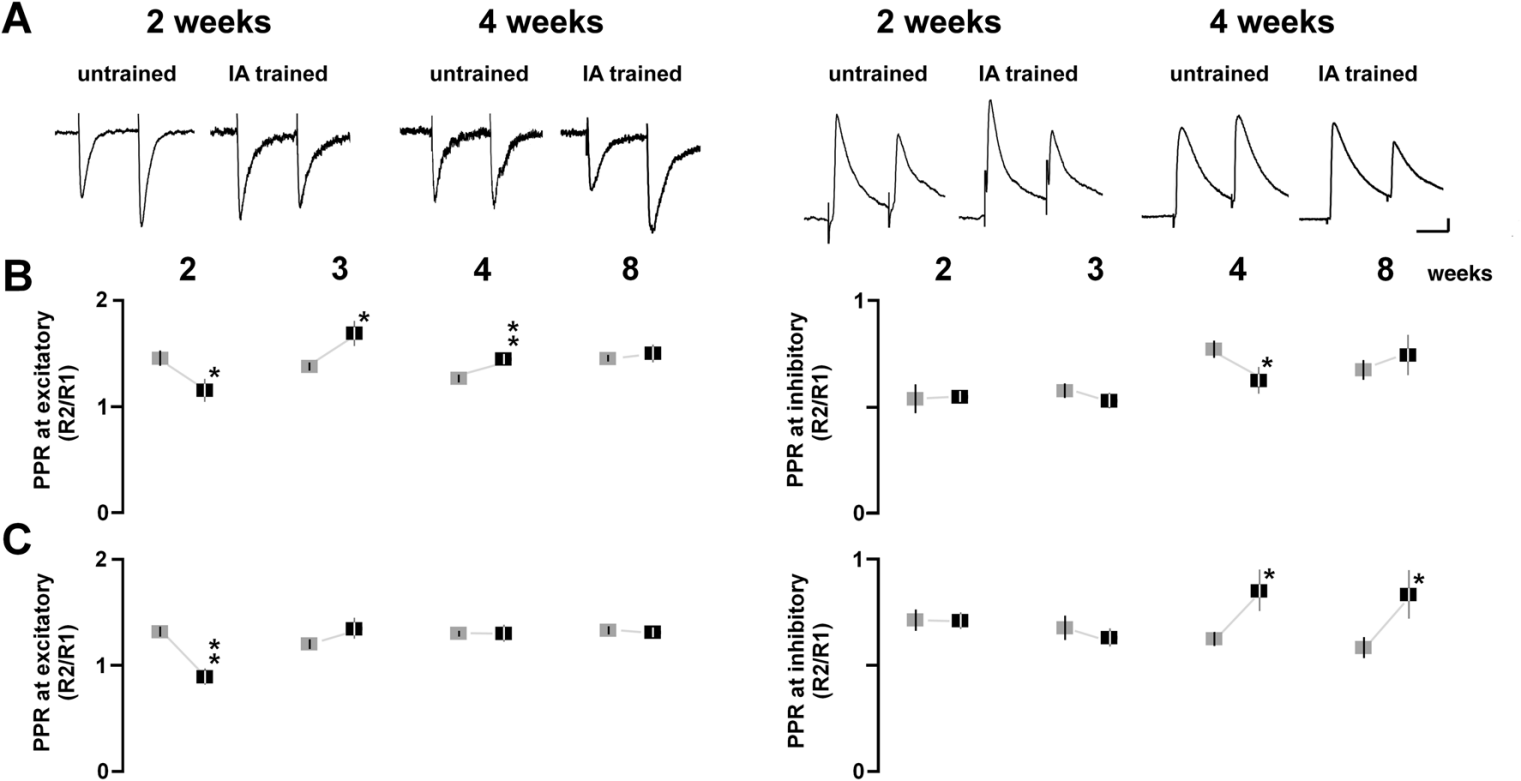
Postnatal development of IA-task-induced presynaptic plasticity. (**A**) Representative traces of AMPA (left) and GABA_A_ (right) receptor-mediated paired-pulse responses stimulated apical dendrites of CA1 neurons. (**B**) Postnatal development of the ratios by apical dendrite stimulation in untrained (gray) and trained (black) rats. (**C**) Postnatal development of the ratios by basal dendrite stimulation in untrained and trained rats. The numbers under the graphs indicate the number of neurons. The error bars indicate ± SEM. *P < 0.05, **P < 0.01 vs. untrained.

To analyze presynaptic plasticity, we examined the paired-pulse ratio after the training (Fig. 4A). At the excitatory synapses in the apical dendrite, IA training significantly increased the paired-pulse ratio at 3 and 4 weeks, whereas the ratio was decreased at 2 weeks (2 weeks: *t*_62_ = 2.36; *P* = 0.0224; 3 weeks: *t*_62_ = − 2.36; *P* = 0.0214; 4 weeks: *t*_75_ = − 2.79; *P* = 0.0066, Fig. 4B). At the synapses in the basal dendrite, the training decreased the paired-pulse ratio at 2 weeks (*t*_51_ = 4.61; *P* < 0.0001, Fig. 4C). These results suggest that the training increased presynaptic glutamate release at 2 weeks, but decreased it at 3 and 4 weeks.

At the inhibitory synapses in the apical dendrite, the training significantly decreased the paired-pulse ratio at 4 weeks (*t*_65_ = 2.03; *P* = 0.0468, unpaired *t*-test; Fig. 4B). At the synapses in the basal dendrite, the training significantly increased the ratio at 4 and 8 weeks (4 weeks: *t*_64_ = − 2.21; *P* = 0.0335; 8 weeks: *t*_54_ = − 2.09; *P* = 0.0410, unpaired *t*-test; Fig. 4C).

Early-life physiological functions may affect latency in the IA task. To rule out this possibility, we evaluated several physiological functions using the visual placing response, open field, light–dark box, flinch/jump, incline, and rotor rod tests. In the visual placing test, one-way ANOVA showed no significant main effect of development (*F*_3,27_ = 0.51, *P* = 0.68), indicating the presence of basic visual function at 2 weeks.

**Figure 5:**
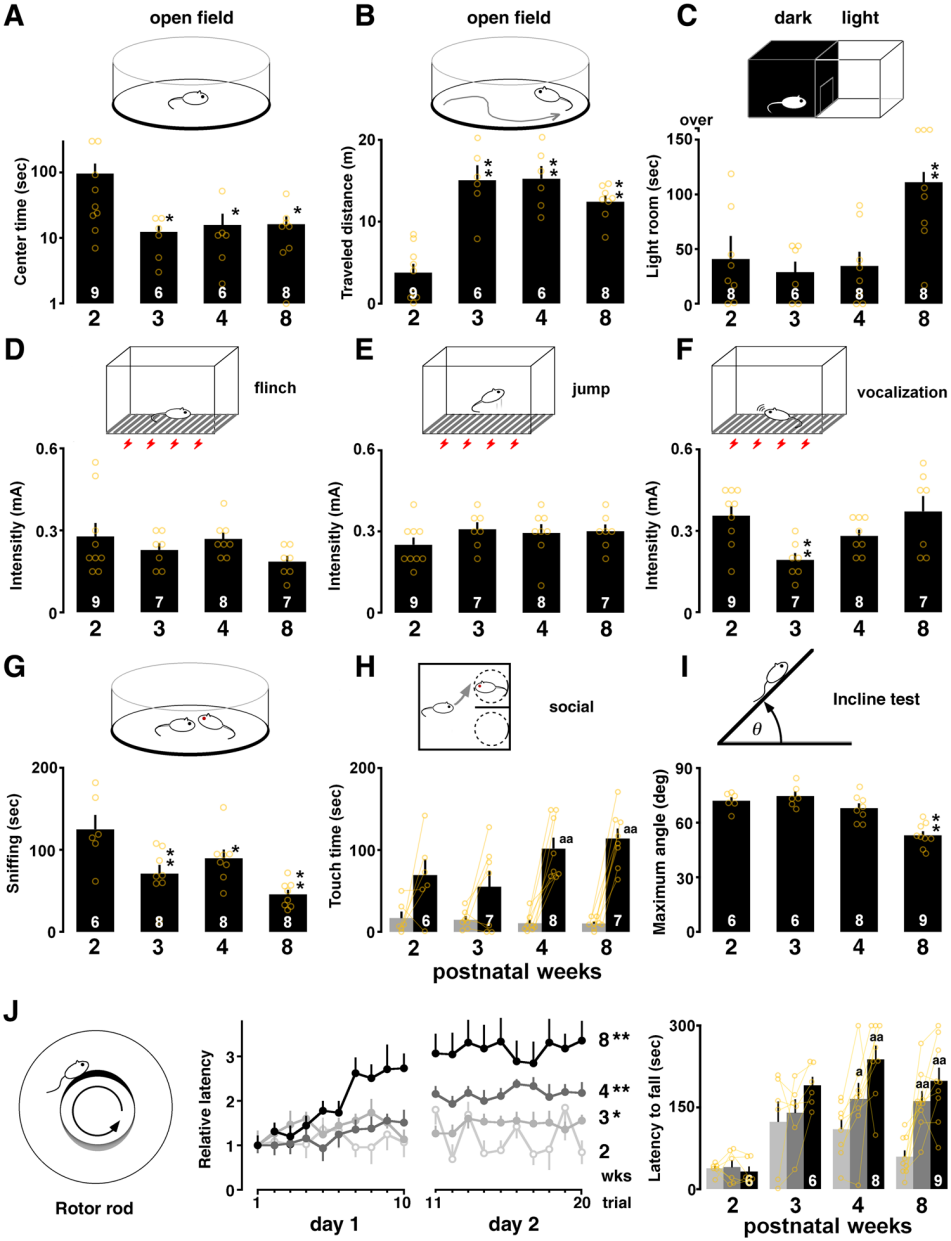
Postnatal changes in the emotional state and sensory/motor functions. (**A**) The latency in the center circle in the open field test was shorter at 3–8 weeks than it was at 2 weeks. (**B**) The traveled distance was longer at 3–8 weeks than it was at 2 weeks. (**C**) The time spent in the lit chamber in the light–dark box test was longer at 8 weeks than it was at 2 weeks. (**D**, **E**) In the sensory test, the current intensity for the flinch or jump was not changed. (**F**) The current intensity for vocalization was low only at 3 weeks. (**G**) The sniffing time of others in social interaction was shorter at 3–8 weeks than it was at 2 weeks. (**H**) Exposure to a social target significantly increased touch time at 4–8 weeks. (**I**) The maximum angle to fall in the incline test was steeper at 2–4 weeks than it was at 8 weeks. (**J**) Relative latency to fall from the barrel plotted for each trial (10 trials/day) at 2–8 weeks. The bar graph showed mean latency at the first trial and the final trial on day 1 (10th trial) and day 2 (20th trial). The number of rats is shown at the bottom of each bar. Data are shown as individual points and are the mean ± SEM. *P < 0.05, **P < 0.01 vs. 2 weeks. ^a^*P* < 0.05, ^aa^*P* < 0.01 vs. training.

To evaluate anxiety and innate curiosity about a novel environment, we used the open field and the light–dark box test (Fig. 5A–C). One-way ANOVA of the open field test results showed a significant main effect associated with the center-circle latency (*F*_3,28_ = 3.81, *P* = 0.0224), and a post-hoc analysis showed that the latency in the center circle of the open field was shorter at 3, 4 and 8 weeks than it was at 2 weeks (Fig. 5A). Conversely, regarding the traveled distance test, one-way ANOVA showed a significant main effect of traveled distance (*F*_3,27_ = 19.397, *P* < 0.0001), and a *post-hoc* analysis showed that the traveled distance in the open field was longer at 3, 4 and 8 weeks than it was at 2 weeks (vs. 3 weeks: *P* < 0.0001; vs. 4 weeks: *P* < 0.0001; vs. 8 weeks: *P* = 0.0002; Fig. 5B). In the light–dark box test, one-way ANOVA showed a significant main effect of development (*F*_3,29_ = 5.97, *P* = 0.0031), and the time in a novel environment was longer at 8 weeks than it was at 2 and 3 weeks (Fig. 5C).

Pain sensitivity was evaluated using the flinch/jump test. One-way ANOVA showed no significant main effect of development (flinch: *F*_3,30_ = 1.47, *P* = 0.24; Fig. 5D; and jump: *F*_3,30_ = 0.94, *P* = 0.4351; Fig. 5E), showing sufficient pain sensitivity at 2 weeks. The current threshold of vocalization at 3 weeks was more sensitive to foot shock than it was at 2 weeks (*F*_3,30_ = 4.73, *P* = 0.0089; 2 vs. 3 weeks: *P* = 0.0037 in post-hoc analysis; Fig. 5F).

To evaluate social behavior, we used the social interaction test (Fig. 5G). One-way ANOVA showed a significant main effect of development (*F*_3,29_ = 8.41, *P* = 0.0005), and the sniffing time of the social target was shorter at 3 and 8 weeks than it was at 2 weeks. By comparing the touching time of a social target with an empty cage, we evaluated social preference. The touching time of social target was longer at 4 and 8 weeks, but not at 2 and 3 weeks (4 weeks: *P* = 0.0006; 8 weeks: *P* < 0.0001, paired *t*-test; Fig. 5H).

We further evaluated motor functions and skill learning performance. In the hanging wire test, one-way ANOVA showed a significant main effect (*F*_3,30_ = 13.140, *P* < 0.0001), and a post-hoc analysis showed that the latency to fall was shorter at 3, 4 and 8 weeks than it was at 2 weeks (3 weeks: *P* = 0.0002; 4 weeks: *P* < 0.0001; 8 weeks: *P* = 0.0001). In the incline test, one-way ANOVA showed a significant main effect (*F*_3,28_ = 18.137, *P* < 0.0001), and a post-hoc analysis showed that the angle to fall was smaller at 8 weeks than it was at 2 weeks (Fig. 5I). In the rotor rod test (Fig. 5J), we measured the latency to falling from the rotating rod, with longer latency considered to indicate better motor performance. For graphic expression, the mean latency at 1st trial was normalized to find the developmental changes. Two-way ANOVA revealed a significant interaction (*F*_57,475_ = 2.39, *P* = 0.0015) and a main effect of developmental stage (*F*_3,475_ = 36.27, *P* < 0.0001) and trial (*F*_19,475_ = 7.76, *P* < 0.0001). Compared with the overall latency at 2 weeks, post-hoc analysis showed that the latency was longer at 3, 4 and 8 weeks (Fig. 5J). By comparing the latency at first trial, we observed significant improvement of the performance at final trials on day 1 (10th trial) and day 2 (20th trial) at 4 and 8 weeks but not 2 and 3 weeks.

**Figure 6:**
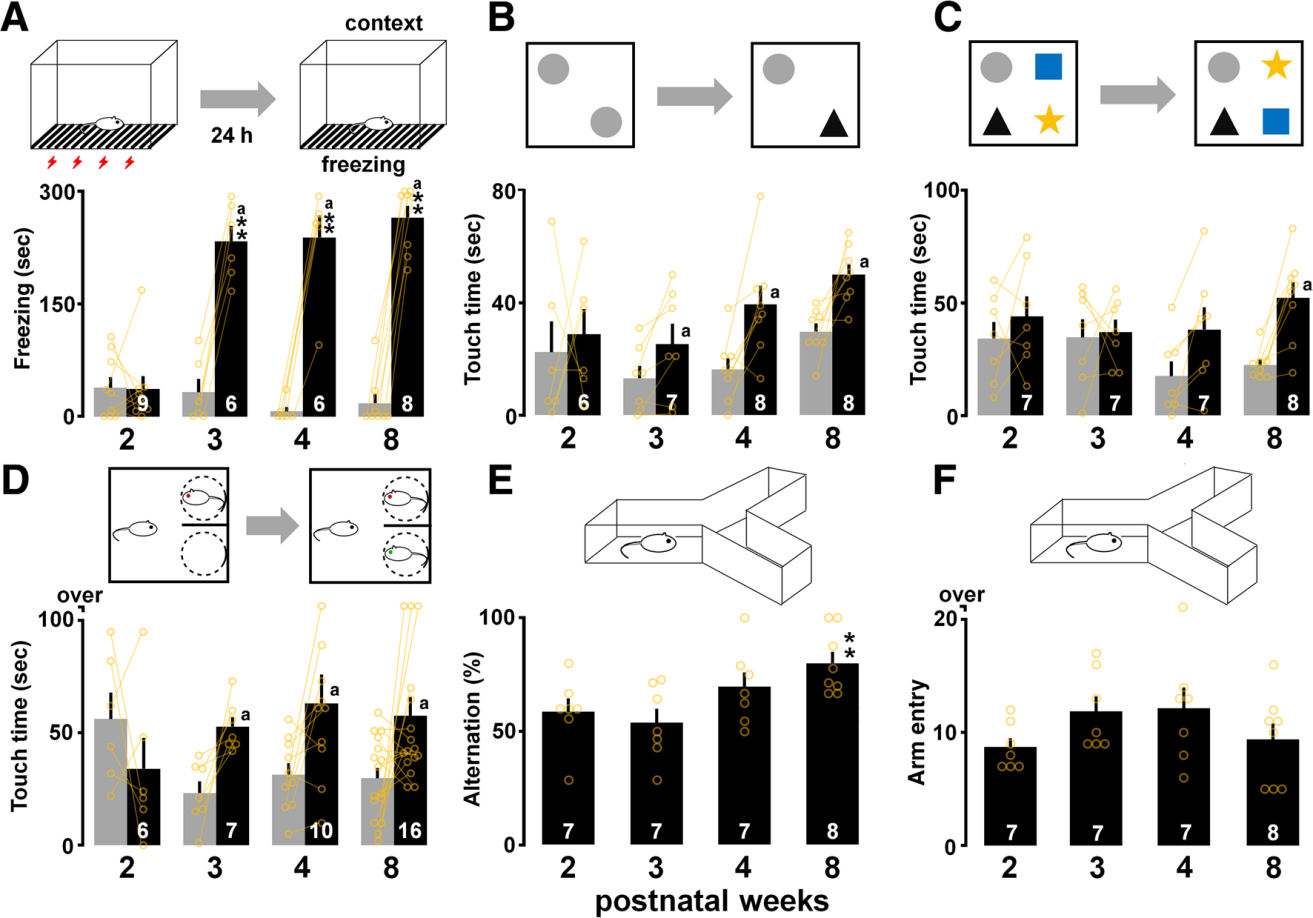
Postnatal changes in other hippocampus-dependent tasks. (**A**) Contextual freezing across training (gray) and testing periods (black). Exposure to context significantly increased averaged freezing time at 3–8 weeks. (**B**) Exploration of familiar (gray) and novel objects (black) in object recognition task. Training increased the touch time of the novel object at 3–8 weeks. (**C**) Exploration of unchanged (gray) and exchanged objects (black) in novel object placement task. Training also increased the touch time of the exchanged objects at 8 weeks. (**D**) Total touch time of familiar (gray) and stranger (black) in social recognition task. Training increased the touch time of the novel target at 3–8 weeks. (**E**) The alternation ratio in the Y-maze test was higher at 8 weeks than it was at 2 weeks. (**F**) Number of total arm entries in the maze. The number of rats is shown at the bottom of each bar. Data are shown as individual points and are the mean ± SEM. *P < 0.05, **P < 0.01.

To analyze further the retention of contextual memory, we compared the performance in the retrieval test at 24 h after the training session in contextual fear conditioning. In contextual fear conditioning, two-way ANOVA revealed a significant interaction (*F*_3,25_ = 46.48, *P* < 0.0001) and a main effect of learning (*F*_1,25_ = 264.28, *P* < 0.0001) and developmental stage (*F*_3,25_ = 22.41, *P* < 0.0001; Fig. 6A). A significant extension of freezing time was observed after 3 weeks of age (3 weeks: *P* < 0.0001; 4 weeks: *P* < 0.0001; 8 weeks: *P* = 0.0001 in post-hoc analysis), which provided further evidence of the existence of a critical period.

To evaluate cognitive functions, we used the object recognition, novel object placement, and social recognition tasks. Prior to the tasks, all groups of rats were checked equal left/right exploration in the sample phase (see Supplementary Tables 1 and 2). In the object recognition task, the training increased the touch time of a novel object at 3, 4 and 8 weeks, but not at 2 weeks (3 weeks: *t*_6_ = − 2.48; *P* = 0.0476; 4 weeks: *t*_7_ = 3.20; *P* = 0.0150, 8 weeks: *t*_7_ = 3.48; *P* = 0.0102, paired *t*-test; Fig. 6B). In the novel object placement task, the training significantly increased the touch time of the exchanged objects only at 8 weeks (*t*_7_ = 4.35; *P* = 0.0033, paired *t*-test; Fig. 6C). In the social recognition task, the training increased the touch time of the novel rat at 3, 4 and 8 weeks, but not at 2 weeks (3 weeks: *t*_6_ = 3.44; *P* = 0.0138; 4 weeks: *t*_9_ = 2.27; *P* = 0.0491, 8 weeks: *t*_15_ = 2.40; *P* = 0.0296, paired *t*-test; Fig. 6D). Thus, neither the novelty nor the changed location of the objects was identified at 2 weeks.

Spatial working memory was evaluated using the Y-maze. One-way ANOVA showed a significant main effect of development (*F*_3,28_ = 4.18, *P* = 0.0158), and the alternation rate was higher at 8 weeks than it was at 2 weeks (Fig. 6E). The total number of arm entries did not change during development (*F*_3,28_ = 1.51, *P* = 0.237; Fig. 6F).

## Discussion

### Developmental changes in memory, sensory, and motor functions

Here, we examined the developmental changes in training-induced synaptic plasticity. At 2 weeks postnatally (postnatal day 16), but not at 3 and 8 weeks (days 22–56), rats could not maintain a memory of IA learning, suggesting an undeveloped contextual memory function in infant rats (Fig. 1 and Ref.^10^). Although rats at 2 weeks showed basic sensory/motor functions, they did not avoid aversive pain by becoming immobilized in the light box. Vision arises around postnatal days 13 and 14^27^, and sufficient pain sensitivity developed before postnatal day 16 in rats (Fig. 5D–F). Regarding motor functions, the incline test showed lower grip performance at 8 weeks (Fig. 5I), but rats at 4 and 8 weeks can learn motor skill in the rotor rod test (Fig. 5J). Moreover, the move into the dark box during IA training session and the immobilization in the dark box in the light–dark box test suggests sufficient motor function for choosing to stay or move (Figs. 1B, 5C). Regarding the emotional state, however, the open field test showed the longest center-circle time at 2 weeks (Fig. 5A). At 8 weeks, rats seemed to be more exploratory (Fig. 5B,C), but also more cautious (Fig. 5A), than they were at 2 weeks. Although the unanxious emotional state observed at 2 weeks could partly have reduced IA latency, the memory deficit may not be attributed to the development of sensory/motor functions.

Thus, since the physiological functions of animals change significantly during development, postnatal age of the animal should be carefully considered when using various rodent models.

### Training-induced plasticity at excitatory synapses

Contextual learning requires plasticity at CA1 synapses at 4 weeks^22–25^; however, a developmental change in learning-dependent synaptic plasticity has not been reported. IA training increased the mEPSC amplitude at 3 and 4 weeks, but not at 2 and 8 weeks (Fig. 2D), indicating the development of training-induced synaptic plasticity at excitatory synapses. Regarding the other evidence of development, naive AMPA receptor densities increased dramatically (184%) during postnatal days 0–10 and were stable during postnatal days 20–30^28^. Moreover, the magnitude of the late-phase LTP at postnatal days 12–15 was almost half that at days 19–35^29^, while early-phase LTP magnitude increased at postnatal days 15–20^30,31^.

In rats at 4 weeks, we previously showed a training-induced increase in the postsynaptic number of AMPA receptor channels without a change in the cation current per single channel in the CA1^25^. Moreover, regarding a causal relationship, we previously demonstrated that bilateral gene expression of GluA1-containing AMPA receptor delivery blockers in the CA1 neurons impairs IA learning^22^, suggesting that newly delivered GluA1-containing AMPA receptors contribute to the formation of contextual memory.

The paired-pulse analysis performed here further revealed presynaptic plasticity at excitatory synapses. In rats at 3 and 4 postnatal weeks, the paired-pulse ratio increased after IA training, indicating a decrease in presynaptic glutamate release (Fig. 4B). Because the mEPSC frequency is used as an indicator of evoked release^32–34^ or the number of functional synapses^35,36^, postsynaptic plasticity may have contributed to the increase in mEPSC frequency observed at 3 and 4 weeks. In our study of the temporal dynamics, presynaptic glutamate release increased at 5 min but decreased at 60 min^24,37^. The late decrease might have been associated with feedback via presynaptic NMDA receptors^38^.

Unexpectedly, training failed to strengthen the excitatory synapses at 8 weeks (Fig. 2D,E). Since IA training is known to induce both synaptic depression and potentiation in adult rats^39^, it is possible that the synapse-dependent occurrence of depression or potentiation counteracted the effect at 8 weeks. The number of synapses^40^ and AMPA receptors^28^ reach a maximum value at 4 weeks and decline slightly at 8 weeks, suggesting that synaptic elimination plays a role in the refinement of network connections^40^. In adult animals, therefore, it is possible that the decrease in E/I balance at 8 weeks of age increased contrast in the firing activity of engram cells, and genetic manipulation to visualize CA1 engram cells may be necessary to detect the spine-specific plasticity after training^41,42^.

Even without engram cell tagging, a 5-min exposure to an emotional experience promotes mean AMPA receptor-mediated current in CA1 neurons of adult mice^43^. We also found a 10-min exposure to an emotional experience, such as restraint stress or contact with a reproductive female, clearly strengthened AMPA receptor-mediated excitatory currents in CA1 pyramidal neurons of adult rats^5^. The result suggesting extent of synaptic plasticity and the created diversity depend on the recent experience in adult animals. It is possible, therefore, that the brief foot shock administered in the IA task may not be sufficient to arouse emotion increasing the mean mEPSC amplitude/frequency at 8 weeks (Fig. 2D,E).

### Training-induced plasticity at inhibitory synapses

IA training also increased the mean mIPSC amplitudes at 4–8 weeks, suggesting a postsynaptic strengthening at GABA_A_ receptor-mediated synapses^23,24^. Because the task also increased mIPSC frequency at 3 and 4 weeks, it may activate silent GABA_A_ synapses, to increase the number of synapses. A genetic deficiency in the GABA_A_ receptor β_3_ subunit and the prevention of GABA_A_ receptor-mediated plasticity in the CA1 both impair contextual learning^23,44^, suggesting a causal relationship between GABA_A_ receptor delivery and learning. Optogenetic manipulation of CA1 neurons has further revealed a timing-specific causal relationship: the inactivation of dendrite-targeting CA1 interneurons during aversive stimuli is sufficient to prevent fear learning^45^. Thus, IA training may trigger GABA_A_ receptor-mediated plasticity to encode contextual memory from the juvenile to adult periods.

We recently found rapid phosphorylation of the Ser^408–409^ GABA_A_ receptor β_3_ subunit immediately after training^24^. In cultured neurons, Ser^408–409^ phosphorylation increases both the amplitude and frequency of mIPSCs by blocking clathrin-dependent endocytosis of the synaptic receptors^46^. Because local interference in Ser^408–409^ phosphorylation of the bilateral CA1 clearly blocked learning- and GABA_A_ receptor-mediated plasticity at CA1 synapses^24^, IA training may lead to rapid phosphorylation of Ser^408–409^ of the GABA_A_ β_3_ subunit, to encode the episodic experience.

### Decrease in excitatory/inhibitory balance in adulthood

Although the training failed to increase the mEPSC amplitude at 8 weeks of age (Fig. 2), the animals successfully maintained the memory of the task (Fig. 1). As for spatial learning, the mIPSC frequency increased with the water maze training^47^, while the mIPSC amplitude increased with the novel object recognition task^5^. This suggests that the pattern of synaptic plasticity differs depending on the type of hippocampal learning.

Because mIPSC amplitude consistently increased at 4–8 weeks of age, the training decreased the excitatory/inhibitory (E/I) balance only at 8 weeks. A decrease in E/I balance is consistently observed in other regions in adult rodents (somatosensory cortex^48^; amygdala^49^. In the prefrontal cortex, the mature GABAergic system of adult animals enables an enhanced inhibitory control, whereas juveniles show a higher E/I balance because of an immature GABAergic system^50,51^. Moreover, optogenetic elevation, but not reduction, of the cellular E/I balance within the mouse medial prefrontal cortex impairs cellular information processing^52^. Although the role of the cellular E/I balance remains unknown in CA1 pyramidal neurons, the decrease of the cellular E/I balance by the mature GABAergic system may play an important role in the creation of synaptic diversity for memory processing.

Shunting inhibition occurs when the reversal potential of the synapse is approximately equal to the resting membrane potential^53^. This occurs when the inhibitory current is carried by Cl^-^ ions or by a particular combination of K^+^ and Na^+^ or Ca^2+^ ions. This type of inhibitory input is silent: it does not change the membrane potential directly, but it can reduce the depolarization^54^ and the mEPSC frequency^55,56^. Although the contribution of shunting inhibition is still unclear, strengthened GABA_A_ receptor-mediated mIPSCs may affect both mEPSC frequency and the E/I balance.

### Critical period and infant-specific synaptic depression

Long-term depression (LTD) has been investigated in neonatal-to-juvenile CA1 neurons for decades^57–60^. LTD induction peaks during days 12–20, and is less effective in mature animals^61^. In addition, at the inhibitory synapses, GABAergic synapses on CA1 pyramidal neurons induce LTD over days 14–21, which involves the GABA_B_ receptor-dependent suppression of adenylyl cyclase^62^. Because the training decreased the frequencies of both mEPSCs and mIPSCs at 2 weeks, but increased them at 3 and 4 weeks (Fig. 2E), the training may decrease the number of functional synapses at 2 weeks, but increase them at 3 and 4 weeks.

The training decreased the mEPSC and mIPSC frequencies at 2 weeks exclusively (Fig. 2E), but increased presynaptic glutamate release without changes in GABA release. Although the underlying mechanism is unclear at inhibitory synapses, activation of AMPA receptors by presynaptic electric stimulation is known to induce silencing in infants, but not juvenile rats, decreasing mEPSC frequency, but not amplitude^63,64^. Since the decrease in mEPSC frequency reflects synaptic silencing at the excitatory synapses^65^, it may provide the first evidence of training-induced synaptic silencing in infants.

As rats at 8 weeks exhibited difficulties in retrieving a memory that was trained at 2 weeks^6^, critical changes in the plasticity response might play a key role in infantile amnesia. The rapid forgetting of contextual memory in infants may be closely associated with the GABAergic system in early development^66,67^. Regarding a causal relationship, bilateral microinjection of a GABA_A_ receptor inverse agonist (FG-7142) into the hippocampus recovered the rapid forgetting of contextual memory on postnatal day 18^66^, whereas the microinjection of a GABA_A_ receptor agonist (midazolam) impaired the retrieval of forgotten memory^67^. These results suggest that tonic GABA_A_ receptor-mediated inhibition in infants may cause the rapid forgetting of contextual memory.

### The development of performance is task-dependent

By analyzing the performance of other hippocampal-dependent tasks (Fig. 6), we identified the task-dependent period of amnesia. Rats learned the IA, fear conditioning, and object recognition tasks after 3 weeks of age; in contrast, they learned the novel object placement task after 8 weeks of age. In the novel object placement task using two objects, rats successfully performed the associative spatial recognition task at postnatal day 30, but not at day 24^68^. Because rats can perform spatial tasks after postnatal days 26–27^69^, the association of place and spatial memory between objects seemed to require a longer developmental period than did the object memory alone.

The number of place cells increases during development^3^. Less than half of CA1 pyramidal neurons have formed in the place field (place-specific firings) at 2 weeks of age in rats, whereas the population has increased by up to 90% in adult animals. Because the targeted disruption of the place field impairs memory-guided spatial behavior^70^, this increase may be closely associated with the development of spontaneous alternation in the Y-maze^71^ (Fig. 6E,F). Moreover, approximately 75% of dorsal CA1 neurons not only process their own location, but also express the location of the other male rat in the same cage^72^, suggesting the existence of a large population of junction-place cells when two adult male rats are housed in the same cage. Although the postnatal change in the population is completely unknown, the development of social behaviors (Figs. 5G,H, 6D) suggest a possible critical period to functionalize the junction-place cells.

## Materials and methods

### Animals

Male Sprague Dawley rats were used in this study (Table 1). After weaning, same-sex groups of 2–3 rats were housed in opaque plastic cages lined with wood chips (length 25 cm; width 40 cm; height 25 cm) at a constant temperature of 23 °C ± 1 °C under a constant cycle of light and dark (lights on: 8:00 a.m. to 8:00 p.m.). We counted the parturition day as postnatal day 1, and litters were reduced to 10 pups per cage on day 2. The pups were weaned on postnatal day 20 or 21 and housed in group cages with free access to food and water. The post-weaning rats were individually housed at least 24 h prior to the experiments, to avoid any episodic experience. Food (MF, Oriental Yeast Co. Ltd, Tokyo Japan) and tap water were available ad libitum in all experimental periods. All animal housing and surgical procedures followed the guidelines of the Institutional Animal Care and Use Committee of Yamaguchi University. All experimental procedures were approved by the Institutional Animal Care and Use Committee of Yamaguchi University (Approval No. 04-S02). the These guidelines comply with the Guide for the Care and Use of Laboratory Animals published by the National Institutes of Health (NIH Publication No. 85-23, revised 1996). The study reported in accordance with ARRIVE guidelines.

**Table 1:** Postnatal groups and body weight.

Postnatal weeks	Postnatal days	Body weight (g)	Number of rats
2	16.0 ± 0.1	34.8 ± 0.6	79
3	21.5 ± 0.1	53.0 ± 0.9	55
4	28.5 ± 0.1	102.5 ± 2.1	67
8	56.2 ± 0.2	306.0 ± 5.5	58

Data are the means ± SEM.

The IA training apparatus (length 33 cm; width 58 cm; height 33 cm) was a two-chambered box consisting of a lit safe side and a dark shock side separated by a trap door (Fig. 1A^22–24^. For training, rats were placed in the lit side of the box, facing the corner opposite the door. After the trap door was opened, the rats could enter the dark box at will. The latency before entering the novel dark box was measured as a behavioral parameter (latency before IA learning, Fig. 1B). Soon after the animals entered the dark side, we closed the door and applied a scrambled electrical foot shock (1.6 mA, 2 s) via electrified steel rods placed in the floor of the box. The rats were kept in the dark compartment for 10 s before being returned to their home cage. Untrained control rats were not moved from their home cages.

Thirty minutes after the procedure described above, the rats were placed in the lit side of the box. The latency before entering the dark box was measured as an indicator of learning performance (latency after IA learning, Fig. 1B).

### Electrophysiological recording of slice-patch clamping

We have previously reported the detailed technical protocol of the slice-patch clamp technique used for analyzing training-induced synaptic plasticity, with a short demonstration movie^73^. Briefly, 1 h after the delivery of the paired foot shock, rats were anesthetized with pentobarbital and acute brain slices were prepared^22,23,25^. We used naive rats as the untrained group, all of which were injected with the same dose of anesthesia in their home cage. For the whole-cell recordings^74^, the brains were quickly perfused with ice-cold dissection buffer (25.0 mM NaHCO_3_, 1.25 mM NaH_2_PO_4_, 2.5 mM KCl, 0.5 mM CaCl_2_, 7.0 mM MgCl_2_, 25.0 mM glucose, 90 mM choline chloride, 11.6 mM ascorbic acid, and 3.1 mM pyruvic acid) and gassed with 5% CO_2_/95% O_2_. Coronal sections (target CA1 area: AP, − 3.8 mm, DV, 2.5 mm, LM, ± 2.0 mm^75^ were prepared (350 μm, Leica vibratome, VT-1200) in dissection buffer and transferred to physiological solution (22–25 °C; 114.6 mM NaCl, 2.5 mM KCl, 26 mM NaHCO_3_, 1 mM NaH_2_PO_4_, 10 mM glucose, 4 mM MgCl_2_, and 4 mM CaCl_2_, pH 7.4) gassed with 5% CO_2_/95% O_2_. The recording chamber was perfused with physiological solution at 22–25 °C.

Although we followed the physiological solution and temperature for many plasticity studies^22,23,76,77^, behaving animals may show different excitatory/inhibitory currents in in vivo brain. In fact, the mEPSC responses are known to depend on Mg^2+^ and Ca^2+^ concentrations in the extracellular fluid^78^, as well as the temperature^79^.

Patch recording pipettes (4–7 MΩ) were filled with intracellular solution (127.5 mM cesium methanesulfonate, 7.5 mM CsCl, 10 mM HEPES, 2.5 mM MgCl_2_, 4 mM Na_2_ATP, 0.4 mM Na_3_GTP, 10 mM sodium phosphocreatine, and 0.6 mM EGTA at pH 7.25). Whole-cell recordings were obtained from CA1 pyramidal neurons from the rat hippocampus using an Axopatch 700 A amplifier (Axon Instruments). The whole-cell patch-clamp data were collected using a Clampex 10.4 instrument, and the data were analyzed using the Clampfit 10.4 software (Axon Instruments).

### Miniature postsynaptic current recordings

We have previously reported the detailed technical protocol of the miniature postsynaptic current recording^23–25^. mEPSCs are thought to correspond to the responses elicited by the presynaptic release of a single vesicle of glutamate. In contrast, mIPSCs are thought to correspond to GABA. Increased mEPSC and mIPSC amplitudes reflect the strengthening of postsynaptic transmission, whereas increased event frequency reflects an increased number of functional synapses or presynaptic release probability.

For the miniature recordings, we added a Na^+^ channel blocker (0.5 μM tetrodotoxin) to the physiological solution. The mEPSCs (− 60 mV holding potential) and mIPSCs (0 mV holding potential) were recorded sequentially for 5 min in the same CA1 neuron. The miniature events were detected using the Clampfit 10.4 software (Axon Instruments), and the events above 10 pA were used in the analysis. We recorded for at least 5 min, to determine the event frequency of mEPSCs or mIPSCs. The amplitudes of the events were averaged to obtain the mean amplitude. Bath application of an AMPA receptor blocker (CNQX, 10 μM) or GABA_A_ receptor blocker (bicuculline methiodide, 10 μM) consistently blocked the mEPSC or mIPSC events, respectively.

#### Paired-pulse stimulation

We have previously reported the detailed technical protocol of the paired-pulse stimulation^24^. To analyze presynaptic plasticity at excitatory synapses, we added 0.1 mM picrotoxin and 4 μM 2-chloroadenosine to the physiological solution and performed paired-pulse stimulation at − 60 mV. To analyze presynaptic plasticity at inhibitory synapses, we added 10 μM CNQX to the perfusate and performed paired-pulse stimulation at 0 mV. To evaluate the paired-pulse ratio from the EPSC or IPSC average, 50–100 sweeps were recorded with paired stimuli at 100-ms intervals. We placed the stimulation electrode in either the stratum oriens (basal) or stratum radiatum (apical), to record evoked somatic currents. The ratio of the second amplitude to the first amplitude was calculated as the paired-pulse ratio^23,80^.

### Self-entropy analysis

Based on the Shannon entropy, we quantified the synaptic diversity by measuring the population differences in mEPSC and mIPSC amplitude or frequency compared with untrained rats^24,25^. We used a standard spreadsheet software (Excel 2010, Microsoft Co., Redmond, WA, USA) to calculate the self-entropy per neuron. First, we obtained four miniature parameters (i.e., mean mEPSC amplitude, mean mIPSC amplitude, mean mEPSC frequency, and mean mIPSC frequency) in individual CA1 pyramidal neurons. Subsequently, we determined the distribution of the appearance probability of four miniature parameters separately using one-dimensional kernel density analysis. The geometric/topographic features of the appearance probability were calculated using a kernel density analysis. Let *X*_1_, *X*_2_,…, *Xn* denote a sample of size *n* from real observations. The kernel density estimates of *P* at the point *x* is given by the following equation:

$${P}_{n}\left(x\right)=\frac{1}{nh}\sum_{i=1}^{n}K\left(\frac{x-{X}_{i}}{h}\right),$$

where *K* is a smooth function called the Gaussian kernel function and *h* > 0 is the smoothing bandwidth that controls the amount of smoothing. We chose Silverman’s reference bandwidth or Silverman’s rule of thumb^81,82^, which is given by the following equation:

$$h = \, 0.{9}An^{{ - {1}/{5}}} ,$$

where *A* = min (standard deviation, interquartile range/1.34). By normalizing the integral value in untrained controls, we identified the distribution of the appearance probability at any point. Subsequently, we calculated the appearance probability at selected points. All data points for probability in untrained and trained rats were converted to self-entropy (bits) using the Shannon entropy concept, as defined in the Information Theory^83^.

To perform calculations using the spreadsheet software, the data for four miniature parameters were summarized in four different sheets, and we obtained the bandwidth (*h*) of individual parameters in the untrained group using the following formula: [= 0.9 STDEV (neuron 1, neuron 2,… neuron *N*)/COUNT (neuron 1, neuron 2,… neuron *N*)^(1/5)^]. Then, using the data from the untrained group, we calculated the distribution of the appearance probability as follows:

- The probability distribution of the first datum for a parameter (neuron 1) was calculated using the formula [= EXP (− (((data of neuron 1 − any point)/*h*)^2/2^))/SQRT (2 × PI())].
- Moreover, the probability distribution of the second datum for the parameter (neuron 2) was calculated using the formula [= EXP (− (((data of neuron 2 − any point)/*h*)^2/2^))/SQRT(2 × PI())].
- Similarly, the probability distribution of the *N* datum for the parameter (neuron *N*) was calculated using the formula [= EXP (− (((data of neuron *N* − any point)/*h*)^2/2^))/SQRT(2 × PI())].
- All probability distributions from neurons 1 to *N* were summed, and the integral value was normalized to 1.

Based on the probability distribution, we calculated the individual appearance probability of all recorded neurons. Subsequently, the appearance probability of the neuron was converted to the self-entropy using Shannon’s formula (= − LOG [appearance probability of the neuron, 2]) (Fig. 3A,B). For graphic expression, the distribution was visualized two-dimensionally in the R software environment (R Foundation for Statistical Computing, Vienna, Austria) (Figs. 2B,C and 3A,B).

### Behavioral test battery

Behavioral tests were performed in the following sequence: open field, object recognition, social preference, social recognition task, social interaction, novel object placement task, light–dark box test, visual placing response test, hanging wire test, Y-maze spontaneous alternation, contextual fear conditioning, and flinch and jump. We used different sampling rats in social preference, social recognition task and social interaction, and different object in object recognition and novel object placement task. The between tests interval was at least 60 min. Rats were habituated to the testing room 30 min prior to testing and the apparatus was cleaned with 70% ethanol between each trial. An additional group of animals was used in the incline and the rotor rod tests.

#### Visual placing response test

To evaluate the sense of sight, we administered the visual placing response test^84,85^. In this test, the rat was suspended by its tail and then lowered toward a black foam plate placed on the front side of its head, without any contact to the vibrissae. Normally, when the head of a rat is lowered to near the edge of the plastic plate, the animal turns its head and trunk and extends its forelimbs to place them on the plate. The success ratio was calibrated to whether the rat successfully placed its forelimbs on the plate^86^.

#### Open field test

To evaluate emotional state and spontaneous locomotor activity, we used the open field test (Fig. 5A,B). The center area (diameter 36 cm) and the peripheral area (diameter 60 cm) of the gray circle floor were lined. After the audio-visual recording, we measured the time spent in the center area and the distance traveled over 5 min^9^.

#### Light–dark box test

To evaluate anxiety and the exploration of a novel environment, we administered the light–dark box test^87–89^. The light–dark box (length 48 cm; width 20 cm; height 23 cm) was constructed of light and dark compartments separated by a sliding door (width 7 cm; height 8 cm). The rats were placed in the center of the dark box and allowed to explore for 5 min. Then, the sliding door was opened, and they explored both boxes for 5 min (Fig. 5C).

#### Flinch and jump test

To evaluate pain sensibility, we performed the flinch and jump test^90^. Rats were placed individually in the fear-conditioning chamber (Fig. 5D–F). The conditioning chamber (length 25 cm; width 31 cm; height 42 cm) was constructed of clear Plexiglass on the top, front, and back. The floor had 18 stainless steel bars (4 mm in diameter; 15-mm spacing), to deliver the scrambled shocks produced by a stimulator (LE100-26 Shocker, Panlab, Cornellà, Spain). After a 3-min period of habituation to the test box, shock titrations continued to increase in a stepwise manner (0.05 mA increments; range 0.05–0.6 mA). In this way, the “flinch” and “jump” thresholds (in mA) were defined for each rat. The interval between shocks was 2 min, and each animal was tested only once at each intensity. The behavior of each rat was recorded through a front digital video recording camera. The “flinch” threshold was defined as the lowest shock intensity that elicited a detectable response. The “jump” threshold was defined as the lowest shock intensity that elicited the simultaneous removal of at least three paws (including both hind paws) from the grid^91^. The “vocalization” threshold was defined as the lowest shock intensity that led to a detectable audible vocalization in response to shock stimuli.

#### Social interaction test

To evaluate the social interaction with an unfamiliar partner (stranger; same strain, sex, and age), we used the social interaction test^92^. Prior to the task, we habituated the rats to an empty open field arena (diameter 45 cm; height 45 cm) for 5 min. Five minutes later, the rats were placed in the center of the arena again. One minute later, we placed an unfamiliar partner in the center of the arena, to assess social interaction for 5 min (Fig. 5G). Sniffing behavior was defined as the animal directing its nose toward or touching the partner.

#### Social preference test

To evaluate social preference, we used a U-field two-choice box^93–95^. The U-field box consisted of two symmetrical rectangular fields that were defined by partitioning an open field (length 45 cm; width 45 cm; height 45 cm) with a wall (length 20 cm; height 45 cm). Prior to the task, we habituated the rats to the U-field two-choice box containing two circular wire empty cages (8 cm in diameter) for 5 min. Five minutes later, the rat was placed in the center of the box again and allowed to freely explore a wire cage containing a social target (stranger; same strain, sex, and age) or an empty wire cage for 5 min (Fig. 5H).

To assess social approach, we measured the time spent touching the social target or empty wire cage in a test phase. Touching behavior was defined as the animal directing its nose toward or its forelimbs touching the wire cage. The apparatus was cleaned with 70% alcohol and air-dried prior to each trial.

#### Hanging wire test

To evaluate basic motor function, we administered the hanging wire test^96^. Rats were placed on a meshed wire, and the wire was turned upside down. We measured the latency until the rats fell to the cage floor lined with soft wood chips.

#### Incline test

We tested the ability of the rats to balance on an angled plywood, which is easy for nails to get caught on, containing a moveable plate with angle adjustment from 0° to 90° (Fig. 5I). The rat was placed perpendicularly and the inclination between the inclined plane and the horizontal plane was increased gradually, until the rat could no longer remain on the table for 5 s. The angle was recorded as the maximum value^97^.

#### Rotor rod task

To evaluate change in motor skills, we conducted the rotor rod test (ENV577; Med Associates Inc., St. Albans, VT, USA). The rat was trained for two consecutive days and allowed 10 attempts with a 30 s inter-trial interval for each test (Fig. 5J). The rotor rod was set to increase from 4 to 40 rpm over 5 min, and the duration of rod-riding was recorded. We recorded the average latency to falling from the rotating rod, and considered longer latency as indicative of better motor performance^74^.

#### Contextual fear conditioning

To evaluate the longer retention of contextual memory, we administered contextual fear conditioning using the conditioning chamber described above (Fig. 6A). Under audio-visual recording (IXY3, Canon Inc, Tokyo, Japan), rats were allowed to explore for 3 min. Then, as the aversive unconditioned stimulus, we delivered foot shocks three times (0.8 mA, 2 s duration). Subsequently, the rats were allowed to recover for 30 s in the conditioning chamber and returned to their home cage. Twenty-four hours later, the rats were again placed in the conditioning chamber, and spontaneous behaviors were monitored for a 5-min period. To assess conditioning, we measured the time spent freezing per every 30 s of the testing period. The time spent freezing in the chamber was considered the measure of contextual learning. Freezing behavior was defined as cessation of all but respiratory movements^9^.

#### Object recognition task

In the habituation phase, rats were placed in the center of an empty open field box (length 45 cm; width 45 cm; height 45 cm) and allowed to explore the box for 5 min. In the sample phase, we placed two identical objects in the box (Fig. 6B). We placed the rats in the center of the open field box again and allowed them to explore for 5 min. Five minutes after the sample phase, we exchanged one of the familiar objects with a new object. In the testing phase, we placed the rats in the center of the open field box and allowed them to explore for 5 min. The apparatus was cleaned with 70% alcohol and air-dried prior to the commencement of each trial for each rat. To assess novel object memory, we measured the time spent touching a novel/familiar object during the test phase. Touching behavior was defined as the animal directing its nose toward or its forelimbs touching the object and the minimum touching time for object was set as a criterion of at least one second. Any other touching behavior, such as resting against the object, was not considered as touching^98^.

#### Novel object placement task

Prior to the task, the rats were habituated to the empty open field box for 5 min. In the sample phase, we placed four different objects (A, B, C, and D) in the corners of the arena, respectively (Fig. 6C). The rats were then placed in the center of the arena and allowed to explore for 5 min. During the 5-min delay period, all of the objects were cleaned with alcohol, to remove olfactory cues. In the test phase, the positions of two of the four objects were exchanged, and the rats were allowed to explore for 5 min. The time spent touching the exchanged objects was compared with the time spent touching the unexchanged objects^98^. We chose the exchanged objects randomly and the minimum touching time for object was set as a criterion of at least one second.

#### Social recognition task

Prior to the task, the rats were habituated to the U-field two-choice box for 5 min. Then, in the sample phase, each rat was placed in the center of the box and allowed to explore an unfamiliar target (same strain, age, and sex) placed in one side (Fig. 6D). Five minutes later, the rat was placed in the box again and allowed to freely explore the same target (familiar) or a stranger (novel) for 5 min. To assess social recognition memory, we measured the time spent touching a familiar or novel social target in test phase. Touching behavior was defined as the animal directing its nose toward or its forelimbs touching the object.

#### Y-maze spontaneous alternation

To evaluate spatial working memory, we used a Y-maze apparatus (Fig. 6E,F). The maze consisted of three arms made of gray plastic joined in the middle to form a “Y” shape (MY-10, Shinfactory, Japan). The walls of the arms had an outside slope of 76° (12 cm high), allowing the rat to see distal spatial landmarks. There were no intermaze cues inside the arms.

Prior to the experiment, the rats were allowed to explore the maze for 5 min. Then, 24 h after the habituation, the rats were placed in one arm again and their spontaneous behavior was recorded for 5 min. By analyzing the number and sequence of arms entered, we calculated the score as the number of alternations divided by the total alternations^22^.

### Statistical analysis

We used unpaired *t*-tests to analyze the data for mEPSCs, mIPSCs, and self-entropy. Because the self-entropy data had large variations within the group, we performed *log* (*1* + *x*) transformation prior to the analysis^99^. To analyze the recognition memory tasks (object recognition, novel object placement, social preference, and social recognition tasks), we used paired *t*-tests to compare the time spent in touching novel and familiar targets in test phase. To analyze other behavioral tasks (visual, open field, light–dark box, pain threshold, social interaction, hanging wire, incline and Y-maze spontaneous alternation tests), we used a one-way factorial ANOVA in which the between-group factors were the postnatal weeks. To analyze the developmental change in the IA, contextual freezing, and rotor rod tasks, we used a two-way ANOVA with repeated measures; the between-group factors were the postnatal weeks and within-group factors were trials. Significance was set at *P* < 0.05.

## Supplementary Information

Supplementary Information.

## Data Availability

The entirety of raw data from this study is available from the authors upon request.
