# Supplementary material for: A critical period for learning and plastic changes at hippocampal CA1 synapses
Source: Sci Rep. 2022 May 3;12:7199. doi: 10.1038/s41598-022-10453-z (PMC9065057; doi:10.1038/s41598-022-10453-z)
Supplement: Supplementary file 1 — Supplementary Information. [file 41598_2022_10453_MOESM1_ESM.docx]

**Supplementary Information**

**Title:** A critical period for learning and plastic changes at hippocampal CA1 synapses

**Authors:** Sakimoto Y, Matsumoto A, Yoshiura D, Goshima M, Kida H, Mitsushima D*

**Affiliations:** Department of Physiology, Yamaguchi University Graduate School of Medicine.

**Suppl. Table 1**  Exploration of familiar and novel objects in sample phase of object recognition task.

------------------------------------------------------------------------------------------------------

Postnatal weeks Familiar Novel (to-be-changed) *P*-value

------------------------------------------------------------------------------------------------------

2 24.5 ± 9.5 19.3 ± 4.6 0.6142

3 35.3 ± 14.3 20.0 ± 3.7 0.3416

4 26.6 ± 4.6 27.3 ± 3.8 0.8598

8 46.3 ± 5.6 33.8 ± 4.4 0.1308

------------------------------------------------------------------------------------------------------

Data are the means ± SEM.

**Suppl. Table 2** Exploration of familiar and novel objects in the sample phase of novel object placement task.

------------------------------------------------------------------------------------------------------

Postnatal weeks Familiar Novel (to-be-changed) *P*-value

------------------------------------------------------------------------------------------------------

2 55.6 ± 10.7 41.1 ± 14.3 0.5589

3 58.7 ± 15.2 34.3 ± 6.9 0.1731

4 34.9 ± 6.7 43.3 ± 11.9 0.3240

8 37.5 ± 3.3 43.8 ± 4.7 0.2137

------------------------------------------------------------------------------------------------------

Data are the means ± SEM.


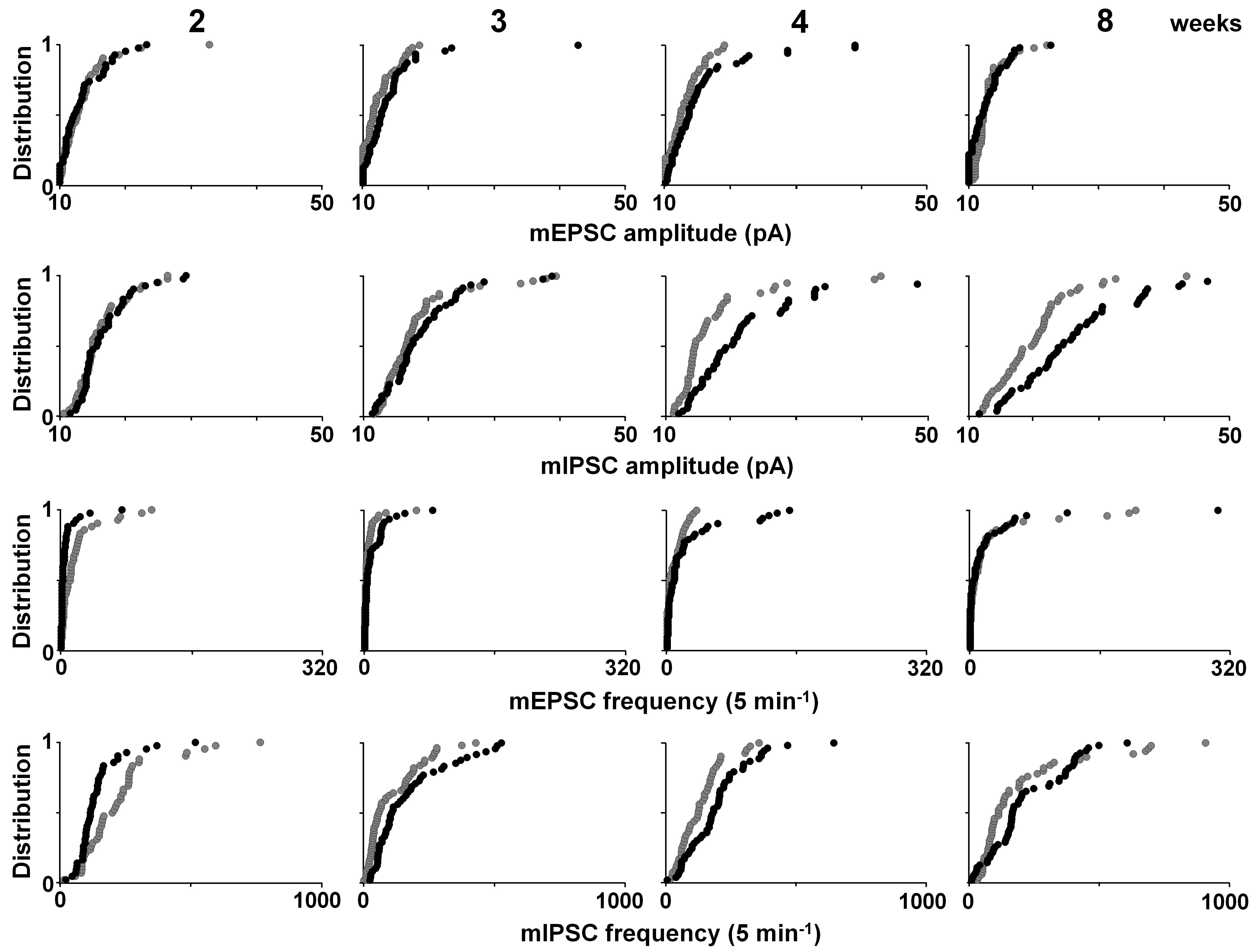


**Suppl. Figure 1** Cumulative distribution of mEPSC amplitude, mEPSC frequency, mIPSC amplitude, and mIPSC frequency in untrained (gray) and trained (black) rats. Individual dots indicate mean levels of individual CA1 neurons. The data were also shown in Figure 3.
